# 3D imaging for dental identification: a pilot investigation of a novel segmentation method using an intra oral scanning device

**DOI:** 10.1007/s12024-025-00992-y

**Published:** 2025-03-18

**Authors:** Harry Perkins, Adam B. Rohrlach, Toby Hughes, Alex Forrest, Denice Higgins

**Affiliations:** 1 Adelaide Dental School, Faculty of Health and Medical Sciences, University of Adelaide, Level 3 Helen Mayo South, 30 Frome Road, Adelaide, Australia; 2 School of Computer & Mathematical Sciences, Department of Archaeogenetics, University of Adelaide, Adelaide, Australia; 3 Max Planck Institute for Evolutionary Anthropology, Leipzig, Germany; 4 Queensland Health Forensic and Scientific Services, Coopers Plains, Australia; 5 University of Queensland School of Dentistry, Herston, Australia; 6 Griffith University, Nathan, Australia

**Keywords:** Dental identification, 3D dental imaging, Segmentation, Identification tools, Forensic odontology, Laser scanning

## Abstract

**Introduction:**

Forensic dental identification relies on the comparison of antemortem and postmortem dental records. 3D dental imaging presents the potential for detailed anatomical features of teeth to be quantified between individuals in automated identification tools. This study introduces a novel segmentation method to simultaneously remove extraneous data from two images reducing processes and time required during 3D dental image comparisons, and tests this against existing approaches to better understand segmentation techniques for forensic purposes.

**Methods:**

Six volunteers had both digital and stone cast full arch dental models created. The casts were scanned and digitized with an intra oral laser scanner, and five different segmentation methods were then applied to all images. Segmented images were compared via a method for aligning 3D images for possible matching (same person) and non-matching (different person) pairings.

**Results:**

All segmentation methods removed adequate excess materials to provide consistent repeated outcomes in the comparison process, with the novel segmentation method showing equivalent outcomes with existing methodologies. The findings highlight the importance of understanding the process of segmentation in distinguishing between 3D dental imaging and underscore the potential of 3D imaging technologies in forensic odontology.

**Conclusion:**

The study demonstrates the efficacy of a new segmentation method in forensic dental identification, offering a faster approach; calling for further validation of these methods within a legal framework.

## Introduction

Forensic dental identification plays a pivotal role in the scientific determination of individual identities in cases involving deceased individuals when other methods may not be feasible due to the condition of remains [1–3]. To determine the likelihood that two sets of data are from the same individual, potentially individualizing characteristics of a person's dentition and oral structures are compared across data collected before (Antemortem, AM) and after (Postmortem, PM) death. Data examined generally includes the shape, size, position, and interrelations of both orofacial anatomical features and dental restorative work present and protected within teeth [3, 4]. Differences between AM and PM data of the same individual may be seen because of a lack of information in one or both datasets and reconciled as being due to developmental, pathological, or interventional changes over time [5, 6].

Traditionally, dental identification has relied on two-dimensional (2D) radiography, despite its limitations in accurately portraying three-dimensional (3D) structures. Increases of advanced 3D imaging technologies in both clinical and forensic practice offers a significant leap forward [7, 8]. These technologies not only capture and provide 3D image files of detailed anatomical information but also pave the way for automated identification tools, potentially reducing the subjectivity and bias associated with manual comparisons [9–11]. Algorithms such as iterative closest point (ICP) registration have been used by software programs to compare 3D dental images represented as discrete data points within 3D space called ‘point clouds’ [12–14]. This ICP process ‘registers’ (superimposes within 3 dimensions) one 3D image onto another and based on the best fit of these data points iteratively rotates and translates the two images until a minimum difference between them is achieved [15, 16]. This provides not only automatic superimposition of images for visual inspection but an objective difference between 3D dental images expressed as a Root Mean Square (RMS) value [17, 18]. It has been demonstrated that two compared 3D dental images from the same individual have a smaller RMS value than images from different people [19–21]. Although no current threshold of expected values for matching and nonmatching dentitions exists, a quantified result from 3D image superimpositions would provide an objective analysis to forms opinions on aiding future dental identifications [22]. Any new technique or methodology for identification must be validated in order to be accepted in a legal context [23, 24]. Potential technical and human errors within an ICP based tool for human identification casework have not been fully explored.

A critical aspect when comparing 3D dental images is to manage disproportionate extraneous information, which can inflate the measure of difference between the two images [25, 26]. Extraneous data, including gingiva and other oral soft tissues, are frequently captured inadvertently during 3D imaging. These tissues are less consistent than dental hard tissues and hence, they are typically excluded from forensic odontology comparisons [6, 27]. Historically, two primary methods for removing this unwanted material from 3D images have been employed: manual reduction along gingival structures, and planar slicing [28–30]. Planar slices are faster to perform and typically leave interdental papillae intact by creating a single cut to the image in one dimensional plane. Manual reductions are more thorough, taking considerably longer, with precise removal along gingival margins into interdental spaces eliminating all but the intraoral hard tissues. The key distinction between these methods is the extent of soft tissue removal as seen in Table 1. Despite their application, a comprehensive evaluation comparing these approaches remains absent from the literature [31–33]. Proprietary software automating this manual reduction is available with high start-up and ongoing licensing fees. In these methods, segmenting images individually before comparison is required, adding to the procedural complexity and time requirement. A simpler method enabling more efficient identification comparisons without compromising accuracy could significantly benefit dental identification, particularly Disaster Victim Identification (DVI) efforts in large-scale incidents [34].

**Table 1:**
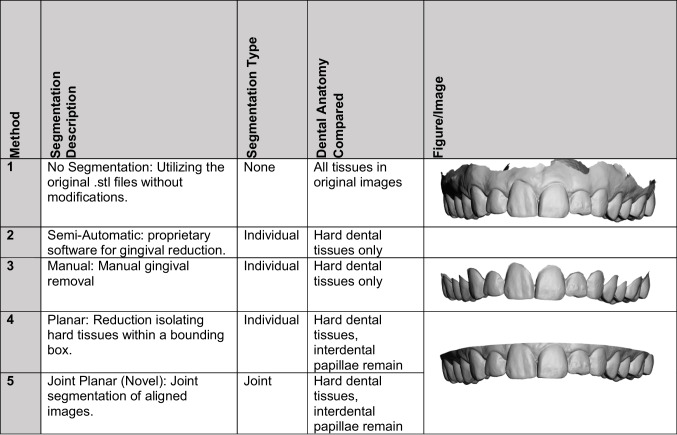
Segmentation methods applied

This study introduces a novel method that streamlines the comparison process by optimally superimposing the two untrimmed images and performing a concurrent planar slice on the combined image with a subsequent superimposition of the residual information. This approach aims to reduce the number of steps and time involved compared to traditional segmentation methods where images are processed separately. We hypothesize that this new method will yield outcomes comparable to existing techniques with the potential for reduced human error and enhanced applicability to automation processes. We compare this novel segmentation method to existing methods and examine the outcomes for both known matching and nonmatching datasets – a key aspect for forensic identification methods.

## Materials and methods

This pilot study is an observational, analytical, cross-sectional design. It provides a snapshot comparison of various segmentation techniques within a controlled framework, including a novel method not previously reported in the literature, applied to 3D dental imaging for forensic identification. Eligibility criteria included adults with complete upper and lower dentitions, free of significant dental restorations or orthodontic treatments. Informed consent was obtained from all participants in accordance with ethical approval (HREC_2021-126). The study aimed to evaluate whether outcomes and errors within segmentation methods differed between existing approaches and a novel technique that jointly segments images, thereby reducing the time and processes required.

### Image collection

Dentitions were digitally captured directly using a Trios intra-oral scanner (Trios 4, software v 1.7.9.1 (3Shape, Copenhagen, Denmark). Dental alginate impressions were taken on the same day, from which stone casts were prepared and subsequently scanned with the same device. The resulting images were exported as.stl files, yielding two full arch scans per jaw—totaling 24 scans. Direct scans were used as antemortem (AM) images (*n* = 12) and scans of casts as postmortem (PM) images (*n* = 12).

### Segmentation methods and operational procedures

Five segmentation methods were applied to each of the 3D dental images, categorized by whether segmentation was performed individually on each image before initial ICP registration (Pre-ICP Registration) or jointly on both images after initial ICP registration (Post-ICP Registration) (Table 1).

All segmentation methods reduced initial images to include dental hard tissues, being the second molar to second molar for each arch (14 teeth in total for each upper and lower arch).

Method 1 serves as a non-segmented baseline for comparison providing a control for evaluating how much segmentation impacts matching accuracy.

Semi-automatic gingival reduction along the gingival margin (Method 2) was executed with proprietary software (3Shape OrthoAnalyser Software). Manual gingival reduction (Method 3) replicated this process manually using MeshMixer (3D software, Autodesk, San Rafael, CA, USA) [35]. Individual planar slices (Method 4) were performed with the cross-section tool in CloudCompare software (v2.6.0, EDF, Paris, France) [36, 37]. For these methods, the two compared images were segmented individually before ICP registration.

Method 5 (Novel Joint Planar) introduces a novel approach. Image files were imported and registered by ICP algorithms with a single joint planar slice performed concurrently on the superimposed images, followed by a secondary ICP registration (CloudCompare software, v2.6.0, EDF, Paris, France) [36, 37]. This was performed thrice and repeated after one month to test for intra-operator consistency in the process. A second operator replicated the process a further three times to assess inter-operator variability.

For the individual segmentation methods (2–4) the PM images were segmented three times, repeated after one month, and by a second operator to create a comparable dataset with only one segmentation variable.

**Fig. 1:**
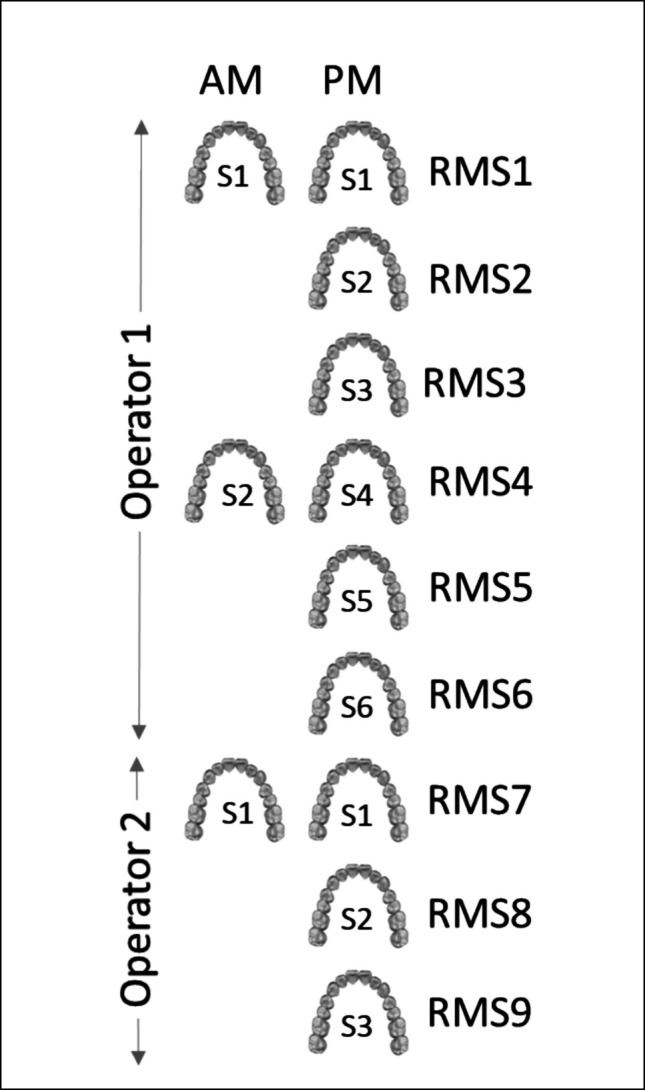
Segmentation methods and comparison groupings. This figure outlines the segmentation process and comparisons performed across all methods. Segmentations (S1-S6) and comparisons (RMS1-RMS9) were applied to both antemortem (AM) and postmortem (PM) full-arch scans for six participants. Matching groups (1v1, 2v2, 3v3, 4v4, 5v5, 6v6) represent comparisons of the same individual's AM and PM scans, while non-matching groups (1v2, 1v3, 1v4, 1v5, 1v6, 2v3, 2v4, 2cv5, 2cv6, 3v4, 3v5, 3v6, 4v5, 4v6, 5v6) compare scans from different individuals. A total of 1,800 comparisons were conducted across all methods

ICP registrations of all matching and non-matching images were performed with a dataset of 1,800 comparisons across all methods and pairings (Fig. 1). Outcome variable RMS values and standard deviations, a common output in the literature, were recorded [22]. Higher values being associated with greater differences between compared 3D dental images.

### Statistical analysis

Statistical evaluations included the development of a predictive RMS mixed effects regression model, incorporating comparison type and segmentation method as predictors to account for variability between individuals and segmentation methods. [38]. A Box-Cox transformation was applied to refine the regression model by addressing non-linearity and ensuring a normal distribution of residuals, enhancing model reliability [39]. This technique adjusts data to better fit the assumptions of parametric statistical tests by identifying the optimal power transformation (e.g., logarithmic or square root) for the dataset. Nested model comparisons were performed using ANOVA, with a p-value cutoff of α = 0.05 to evaluate segmentation effects. To perform pairwise comparisons of the performance of the methods, we used the estimated marginal means as calculated by the emmeans package using a Bonferroni p-value adjustment [40, 41]. All statistical analyses were performed using R statistical programming language, and visualizations were created using ggplot2 [42, 43].

## Results

**Fig. 2:**
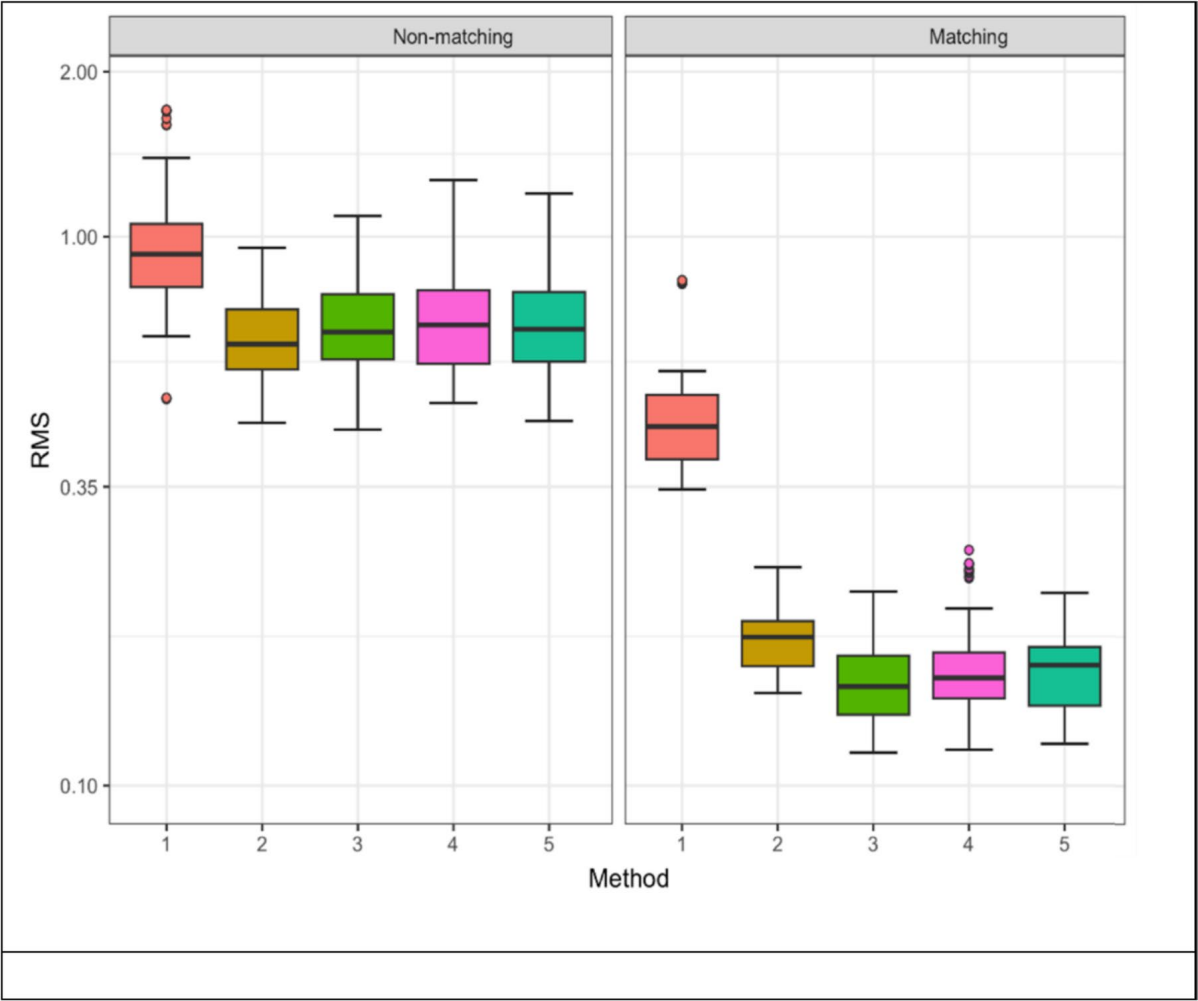
Root mean square values for all groups. Box plots illustrating RMS (Root Mean Square) values for matching and non-matching pairs across the five segmentation methods. Segmentation methods 1–5 for both matching and non-matching groups were compared. Method 1 – no segmentation, Method 2 – Semi-automatic, Method 3 – Manual, Method 4 – Planar, Method 5 – Joint Planar. No overlap was observed between RMS distributions for matching and non-matching groups

All segmentation methods (Methods 2–5) gave lower RMS values for matching individuals compared with no segmentation (Method 1) but differences were not significant for non-matching comparisons (Fig. 2).

No method of segmentation yielded lower RMS values for nonmatching pairs than those observed for known matching pairs. A logistic regression analysis identified an RMS cutoff value of approximately 0.3537 that predicts with 100% accuracy the matching vs. non-matching teeth for all methods except no segmentation (Method 1), emphasizing the efficacy of these segmentation approaches in distinguishing between matching and nonmatching pairs.

The effect of the operator was marginal and subsequently this was dropped from the analysis (*p =* 0.06835) while the participants remained a significant random factor (*p <* 2.2 × 10^–16^). Both matching status and method emerged as significant predictors of RMS values (*p <* 2.2 × 10^–16^).

Within matching cases, estimated marginal means identified Method 1 as significantly less precise than other methods, with Method 2 showing a higher RMS compared to all other methods except Method 1. Conversely, Method 3 demonstrated a lower RMS compared to Methods 4 and 5, establishing Manual segmentation as statistically superior due to the lower RMS values. Despite the quantitative differences, qualitative analysis indicated that all methods achieved 100% accuracy in predicting tooth matches using the above cut off.

**Fig. 3:**
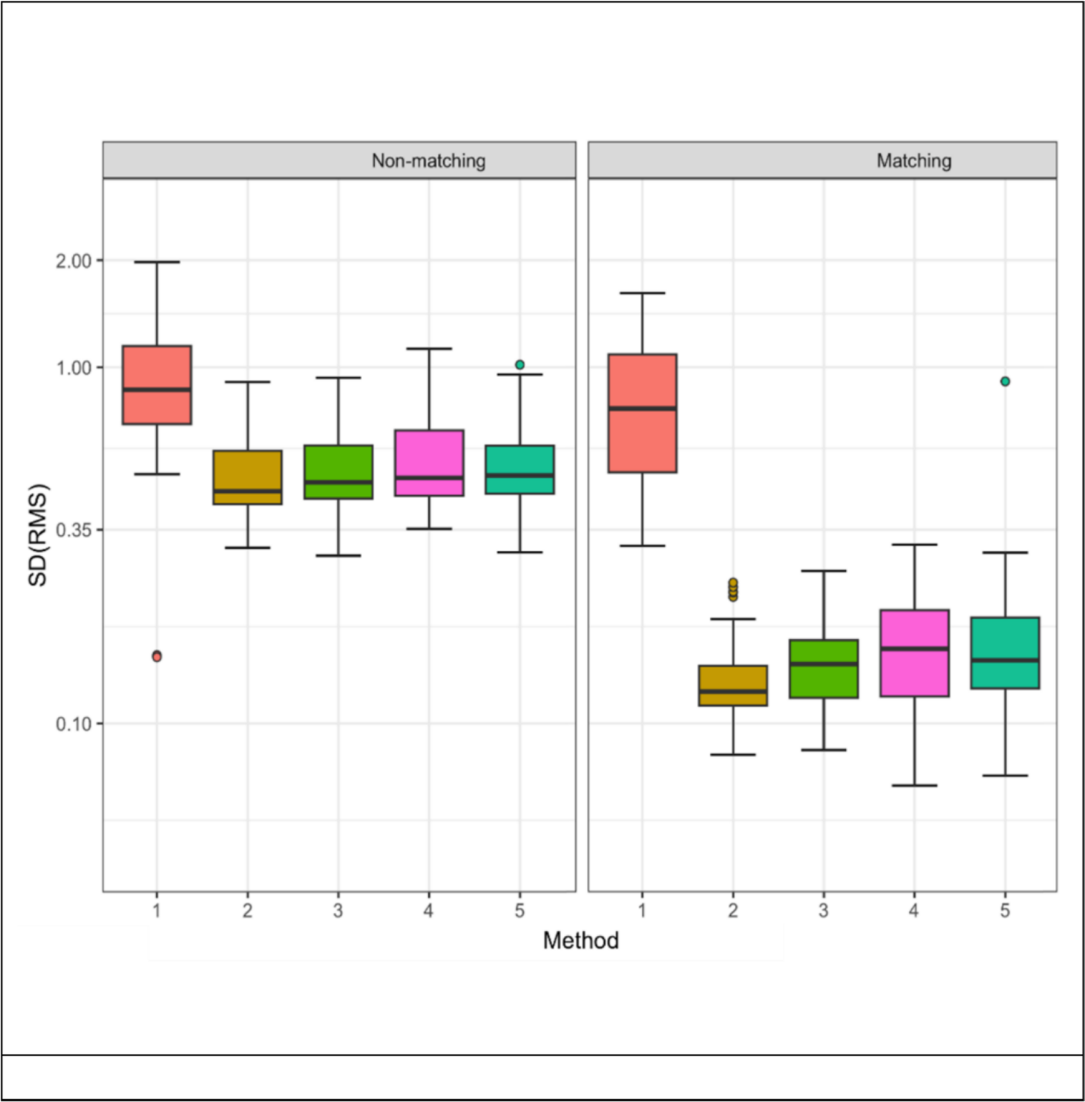
Root mean square standard deviation values for all groups. Standard Deviation (SD) distributions of Root Mean Square (RMS) values for each segmentation method (1–5). Methods 4 (planar segmentation) and 5 (novel joint segmentation) demonstrated greater spread in RMS values, particularly for matching groups, indicating increased variation in segmentation outcomes. Despite this variability, all segmentation methods maintained 100% accuracy in distinguishing between matching and non-matching pairs

Analysis of variance in standard deviations (Fig. 3) revealed notable variability among segmentation techniques. Operator 1 exhibited greater variability in RMS values for matching teeth but less variability for non-matching teeth. Methods 4 and 5 showed broader standard deviation distributions, suggesting increased variability in RMS outcomes. However, this variability did not compromise the predictive accuracy of these segmentation techniques for distinguishing between matching and non-matching pairs.

## Discussion

The evaluation and comparison of dental hard tissues plays a critical role in forming reliable forensic odontology identification opinions. By isolating intraoral dental hard tissues in 3D imaging from surrounding soft tissue anatomy through segmentation, focused comparisons can be achieved, minimizing discrepancies caused by variable capture ranges, soft tissue changes over time, or postmortem alterations. The primary goal of this pilot study was to evaluate and compare various segmentation methods, including a pioneering approach, to enhance the process of dental comparisons in 3D imaging. Additionally, this study aimed to better understand errors within and between segmentation techniques, contributing to the growing body of evidence in 3D forensic odontology and supporting the validation of these methods in forensic contexts.

As corroborated in this study, segmentation of extraneous data allows for a better fit (lower RMS) between two 3D images of the same individual than no segmentation [44]. However, no previous studies have investigated how various segmentation approaches affect the comparison of images from two different individuals. The consistent performance of all segmentation methods, as demonstrated in both matching and non-matching scenarios, affirms the use of both planar and gingival margin reduction strategies in forensic investigations. The results also suggested that both individual and joint planar reductions have comparable outcomes, with the novel joint approach having fewer stages and reducing time to perform.

Although it is widely accepted and supported by this study that segmentation is advantageous, to what extent soft tissue is a hinderance is uncertain. The results seen when comparing methods with and without inclusion of interdental gingival papilla would suggest this structure has no or little impact on the comparison outcome. This observation aligns with the understanding that the core identifiers in forensic dental identification lie within the hard tissues of the teeth [29, 45]. It was interesting to note that these planar reductions were found to have an increased SD within tested methods suggesting increased error in these processes when selecting anatomical landmark features (Fig. 3). In a real-world scenario greater difference would be likely between AM and PM images due to growth and development, disease processes, dental interventions and postmortem changes. While these changes need to be considered when performing a reconciliation, they would be an equally important consideration whatever methodology is used. Segmentation of 3D models will ultimately need to take this into consideration. Considering the high variability in AM and PM data in forensic odontology casework, developing adaptable evidence-based identification methods is critical for both matching and non-matching datasets.

Although intra and inter-operator differences were present, no significant impact on the RMS outcomes was identified. These differences were likely from residual tooth structure available after selection, or from incorrect file placement during ICP registration, although in previous studies this has been found to have limited effect [46]. While this variability appeared to have a limited impact on the outcome, it signals potential issues warranting further investigation with specialized skill sets and experience in these procedures required.

Currently segmentation methods often rely on proprietary software which can have startup and ongoing licensing costs. Customized open-source software solutions could reduce reliance on such tools [47]. The joint segmentation and planar alignment method demonstrated in this study is demonstrated as effective as conventional techniques while being more time-efficient and fully automatable. Emerging machine learning and deep learning approaches offer potential for excluding non-dental structures from 3D models, further streamlining identification and reducing manual effort and error [48, 49].

While the same scanner was used here, variability in 3D data collection systems between AM and PM datasets, requires further investigation. Comparisons may have been impacted by the inclusion of stone casts into the data pool along with direct intra oral scans, however, this variance in dental data replicates the possible differences in forensic casework presentations. Forensic casework often involves AM images from diverse sources, introducing challenges such as differences between imaging creation and 3D formats or extended time differences between AM and PM imaging. Understanding the impact of AM data recency on biological differences with larger sample sizes remains a key area for future research. Similarly not including dentitions with dental restorations and orthodontic treatments was a limitation, reducing the generalizability of results. It should be noted that oral health trends and evolving management philosophies focusing on preventative minimal invasive techniques may result in future dentitions without restorative interventions [50]. A key focus of future identification methodologies is to better understanding the anatomical variation between individuals without interventions [51]. Examination of a larger data set may improve the robustness of this investigation, however, considering this study’s design, further participants would have significantly increased the number of comparisons between unmatching data sets and hence time needed. Our research did not cover DICOM files or CT images, which can be converted into relevant stl files for this process and being prevalent in PM settings represent critical areas for future exploration [52, 53]. The capability of these 3D radiographic imaging modalities to capture deeper anatomical structures than laser scanning highlights the need to broaden techniques beyond dental models to encompass craniofacial structures. This need is supported by emerging research in fields such as frontal sinus overlap and volumetric segmentation of the nasal airway [35, 40], underlining the interdisciplinary nature of forensic identification.

While this pilot study confirms known outcomes, its unique contribution lies in applying these methods to 3D forensic odontology and demonstrating their comparative efficacy. It also identifies areas for improvement and emphasizes the need for further research into automated segmentation techniques. Future investigations should focus on developing and testing more sophisticated, automated methods to enhance reliability, reduce manual input, and streamline segmentation of 3D imaging for the forensic identification process.

## Conclusion

This pilot study has highlighted the significance of segmentation techniques in preparing antemortem and postmortem dental 3D images created by an intra oral laser scanner for forensic identification. By introducing a novel joint segmentation method, this research demonstrates a quicker and less labor-intensive approach that offers a valuable improvement over traditional segmentation methods. The application of this new method to full arch dental scans shows promising potential, equaling or even exceeding the performance of established techniques in certain scenarios. As a pilot investigation, these findings provide an essential foundation for future studies and further validation of this method across broader datasets.

## Key points

1. Segmentation methods improve forensic 3D identification
  ○ This study demonstrates that segmentation of 3D dental images significantly improves forensic dental identification by reducing extraneous data and improving alignment accuracy. The findings support the use of segmentation in forensic odontology, particularly in distinguishing between matching and non-matching dental images.
2. Efficiency and accuracy of the novel joint segmentation method
  ○ The novel joint segmentation method introduced in this study performs comparably to existing techniques while reducing processing steps and manual intervention. This approach has the potential to streamline forensic workflows, improving efficiency without compromising accuracy.
3. Future implications for automated forensic odontology
  ○ The results of this study underscore the potential for integrating 3D imaging with automated forensic identification systems. Further validation of these segmentation techniques, particularly with larger and more diverse datasets, could enhance their applicability in legal and forensic casework.

## Declarations

### Design

This pilot study is an observational, analytical, cross-sectional design adhering to STROBE guidelines.
